# Long term impacts of early social environment on chimpanzee white matter

**DOI:** 10.1038/s41598-024-81238-9

**Published:** 2024-12-02

**Authors:** Michele M. Mulholland, Erin Hecht, Michael J. Wesley, William D. Hopkins

**Affiliations:** 1 Department of Comparative Medicine, Michale E Keeling Center for Comparative Medicine and Research, The University of Texas MD Anderson Cancer Center, 650 Cool Water Dr, Bastrop, TX 78602 USA; 2 Department of Human Evolutionary Biology, Harvard University, Cambridge, MA 02138 USA; 3 Department of Behavioral Science, University of Kentucky College of Medicine, Lexington, KY 40536 USA

**Keywords:** Adverse rearing, Brain development, White matter, Chimpanzees, Machine learning, Neuroscience, Development of the nervous system

## Abstract

Early adverse rearing conditions are known to have deleterious consequences on social behavior, cognition, and brain development of both human and nonhuman primates. We analyzed archival diffusion tensor imaging (DTI) data from mother- (MR) or nursery-reared (NR) chimpanzees and used support vector machine learning to determine whether we could retrospectively classify chimpanzees as MR or NR based on white matter fractional anisotropy (FA) decades after their rearing experiences. A significant proportion of chimpanzees were correctly classified as MR and NR based on white matter fractional anisotropy (76.32%; *p* = 0.004). Voxel-based morphometry revealed that MR chimpanzees had increased FA in the splenium/isthmus of the corpus collosum and premotor cortex, while NR chimpanzees had increased FA in the thalamic region, cuneus, and lateral genu of the corpus collosum (*p* < 0.01). A subset of the NR chimpanzees participated in early social interventions, but unlike gray matter, these interventions do not explain misclassification based on white matter. These findings suggest that nursery rearing has long-term effects on both gray and white matter, but that early interventions may ameliorate the effects on gray matter only. Future research should investigate the effectiveness and relative contributions of combined social, cognitive, and nutritional interventions on brain development in nonhuman primates.

## Introduction

Slower growth and protracted infancy are key developmental features of primates, particularly humans^1–5^. The human brain triples in size during infancy and early childhood, mostly due to an increase in white matter as a result of myelination of axon fiber tracks in the cortex^6–9^. Disruption to cortical development during this critical time can produce long-term and potentially detrimental alterations. For instance, acute and chronic childhood stress (e.g., low socioeconomic status, neglect, social deprivation, abuse or trauma, and institutional care) influence brain development as well as social behavior, affect, and cognitive functioning^5,10–17^. For example, total gray and white matter volume, and gray matter thickness are reduced in children who were institutionally reared compared to comparison groups of children raised in home settings^18,19^. Childhood institutionalization also affects white matter organization in the fronto-limbic circuitry, frontal striatal, language and sensory pathways, as well as long association fibers^5^. Isolating the specific adverse effects that influence human behavioral and brain development can be a challenge however, as there are often several confounding or covarying variables associated with adverse environments and experiences. For example, in addition to inadequate social experiences and poor attachment to caregivers, some samples of institutionalized children generally lack adequate healthcare, nutrition, and sensory and cognitive stimulation^5,19–21^.

To isolate the effects of different types of early social adversity and reduce the number of confounding factors, animal models (often rodents and primates) have been developed for more controlled and experimental approaches to hypothesis-testing. Decades of research with nonhuman primates has documented consequences of early social deprivation on cognition, behavior, brain development, and other health outcomes^22–39^. Here, we utilized data from chimpanzees previously reared in research facilities as part of NIH-sponsored chimpanzee breeding programs. These programs increased the number of chimpanzee infants for potential use in a broad range of biomedical research with public health relevance. These chimpanzees were either reared by their mothers in a family setting or raised by human care staff in a nursery setting. Those raised in the nursery were separated from their mother due to either inappropriate maternal behavior^40^ or for experimental purposes.

Previous studies show that mother- and nursery-reared chimpanzees differ significantly on measures of cognition, communication, personality, and social behavior^41–43^. With respect to the brain, mother- and nursery-reared chimpanzees differ in whole brain gray and white matter volume as well as in gray matter thickness within the cortical folds^44^. In addition, chimpanzees can be accurately classified as mother- or nursery-reared based using support vector machine learning on whole-brain gray matter variation^45^ more than 10 years after these rearing experiences. Rather than focus on gray matter, in the current study, we use this same method (support vector machine learning) to determine whether we can also retrospectively classify a subset of these same chimpanzees as mother- or nursery-reared based on white matter variation using diffusion tensor imaging data. Similar to our previous report^45^, here we examined the relationship between misclassification and factors such as sex or participation in early social interventions. In addition, we use voxel-based morphometry to examine white matter variation in relation to early rearing experiences.

## Methods

### Subjects

Data included archival diffusion tensor imaging scans (DTI) from 38 chimpanzees (*Pan troglodytes*). An equal number of nursery-reared (*n* = 19; NR) and mother-reared (*n* = 19; MR) chimpanzees were matched on sex (15 females and 4 males per group) and age at the time of the scan (NR: M = 19.42, SD = 6.64, range = 9–35 years old; MR: M = 18.26, SD = 6.52, range = 11–34 years old). All subjects were housed at the Emory (formerly Yerkes) National Primate Research Center (ENPRC). For this study, NR chimpanzees were operationalized as those separated from their mother within the first 30 days life^40,46^. NR infants were initially housed in incubators and provided standard human infant formula (not supplemented with docosahexaenoic acid/DHA to our knowledge). All NR chimpanzees were cared for by humans until they could sufficiently care for themselves (typically 3 months of age), at which time they were placed with 2–10 same-aged peers in indoor/outdoor housing until they were three years of age^40,46^. At this point, ENPRC integrated NR chimpanzees into larger social groups with mixed-sex adult and sub-adult chimpanzees (4–16 individuals) in indoor/outdoor enclosures. In contrast, MR chimpanzees were defined as those who remained with their mother in ‘nuclear’ conspecific family groups (4 to 16 individuals) in indoor/outdoor enclosures for a minimum of 2.5 years. All work was approved by the Institutional Animal Care and Use Committee at ENPRC and complied with the American Psychological Association guidelines for the ethical treatment of animals. The imaging data are available from the National Chimpanzee Brain Resource at https://www.chimpanzeebrain.org.

### Diffusion tensor image acquisition and post-image processing

Chimpanzees were first sedated for their routine physical exam by a clinical veterinarian, then subsequently anesthetized for the collection of neuroimaging data on 3.0-Tesla scanner (Siemens Trio, Siemens Medical Solutions USA, Inc., Malvern, Pennsylvania, USA). Each subject was first sedated with ketamine (10 mg/kg) or telazol (3–5 mg/kg) and subsequently anaesthetized with propofol (40–60 mg/(kg/h)). Diffusion tensor imaging (DTI) data were acquired using a two-dimensional, echoplanar, spin-echo sequence (TR/TE = 10000/85.3 *ms*, NEX = 1, FOV = 144 × 144 *mm*, matrix = 256 × 256, in-plane voxel dimension = 0.56 × 0.56 *mm*^*2*^, slice thickness/gap = 1.3/0 *mm*, 68 interleaved slices, echo-planar spacing = 816 *µs*). Diffusion tensor imaging (b = 1000 *s/mm*^*2*^) was performed in 72 non-collinear directions with 6 non-diffusion weighted images. Images were acquired in the coronal plane in a space spanning the entire brain. In addition, a co-planar field map was obtained using a gradient echo with images at two echo times: TE1 = 7 *ms*, TE2 = 10 *ms*. Following the scanning protocol, subjects were briefly housed in a single cage adjacent to their social group to safely recover from the anesthesia before returning to their social partners (~ 2–24 h, as determined by the veterinary staff).

Preprocessing of the neuroimaging data included brain extraction, head motion correction, EPI distortion correction, and eddy current distortion correction with FSL (BET, MCFLIRT, TOPUP, and EDDY, respectively;^47–51^) from the FMRIB Software Library (The University of Oxford, Oxford; www.fmrib.ox.ac.uk/fsl). The resulting 4D volumes were further processed using the FDT-DTIFIT function within FSL. A standardized spatial environment for analysis was created from a group-average of individual fractional anisotropy (FA) maps using the *buildtemplateparallel.sh* script included in the open-source registration and normalization software package ANTS (Avants, Tustison, & Song, 2009). Template creation relied on initial linear registration followed by nonlinear registration of all FA maps.

### Support vector machine learning

As in Bennett, Pierre^45^, we used kernel-based support vector machine (SVM) learning that was performed using customized MATLAB (R2015b; MathWorks; Natick, MA; https://www.mathworks.com/products/matlab.html) scripts and standard protocols of the Pattern Recognition for Neuroimaging Toolbox 2 (PRoNTo v2.0; London; http://www.mlnl.cs.ucl.ac.uk/pronto/) for MATLAB^52^. First, the individual template-space FA maps derived from the preprocessing steps above were placed into separate directories corresponding to the MR and NR groups. SVM analysis was performed on isolated white matter volumes to determine the accuracy of in-group classification. SVM analysis included a white matter volume per subject in a leave one subject per group out cross-validation scheme. Within this scheme, 19 iterative cross-validation folds trained on correctly labeled group volumes (89.48% of data) and tested fold classification accuracy on the two left out, unlabeled, subject volumes (10.52% of data). An omnibus SVM model was generated by averaging model parameters from each cross-validation fold, and model accuracy was calculated as follows: $Accuracy=\frac{1}{2}\:(\frac{TC}{TC+FC}+\frac{FC}{TC+FC}),$ where TC and FC are the number of true classifications and false classifications, respectively, considering the classification outcome of each cross-validation fold. The omnibus SVM model was considered significant if it outperformed identically generated models with random, incorrectly labeled group/class assignment more than 950 out of 1000 times (e.g., *p*<0.05). For an SVM model that significantly distinguished between MR and NR groups, model weights for contributing white matter voxels were obtained and displayed in a standardized template space.

### Voxel-based morphometry

Fractional anisotropy (FA) is a measure of white matter integrity which varies with fiber density and axon size and orientation, with higher FA values associated with increased density, larger axon size, and more similar orientation. To test for differences in whole brain FA between NR and MR groups, we performed a voxel-based morphometry (VBM) analysis in SPM12 (Functional Imaging Laboratory, London; https://www.fil.ion.ucl.ac.uk/spm/)^53^ run in MATLAB on a Macintosh computer. Previous studies have used VBM with SPM to examine fractional anisotropy reductions in human clinical populations (e.g.,^54–56^). For this analysis, we imported the preprocessed, template-space FA maps for each subject and ran t-test contrasts comparing the two early rearing conditions (NR or MR) while controlling for sex and age. The two contrasts were used to detect differences in FA intensity on a voxel-by-voxel basis between groups. To account for multiple comparisons and reduce the likelihood of Type I errors, the significance threshold was set at *p* < 0.01 uncorrected with a minimum cluster threshold of 50 voxels. After identifying regions which differed between mother- and nursery-reared chimpanzees, we visualized these regions by overlaying the resulting VBM components on the standardized chimpanzee template brain using Analyze 11.0 software.

## Results

**Fig. 1:**
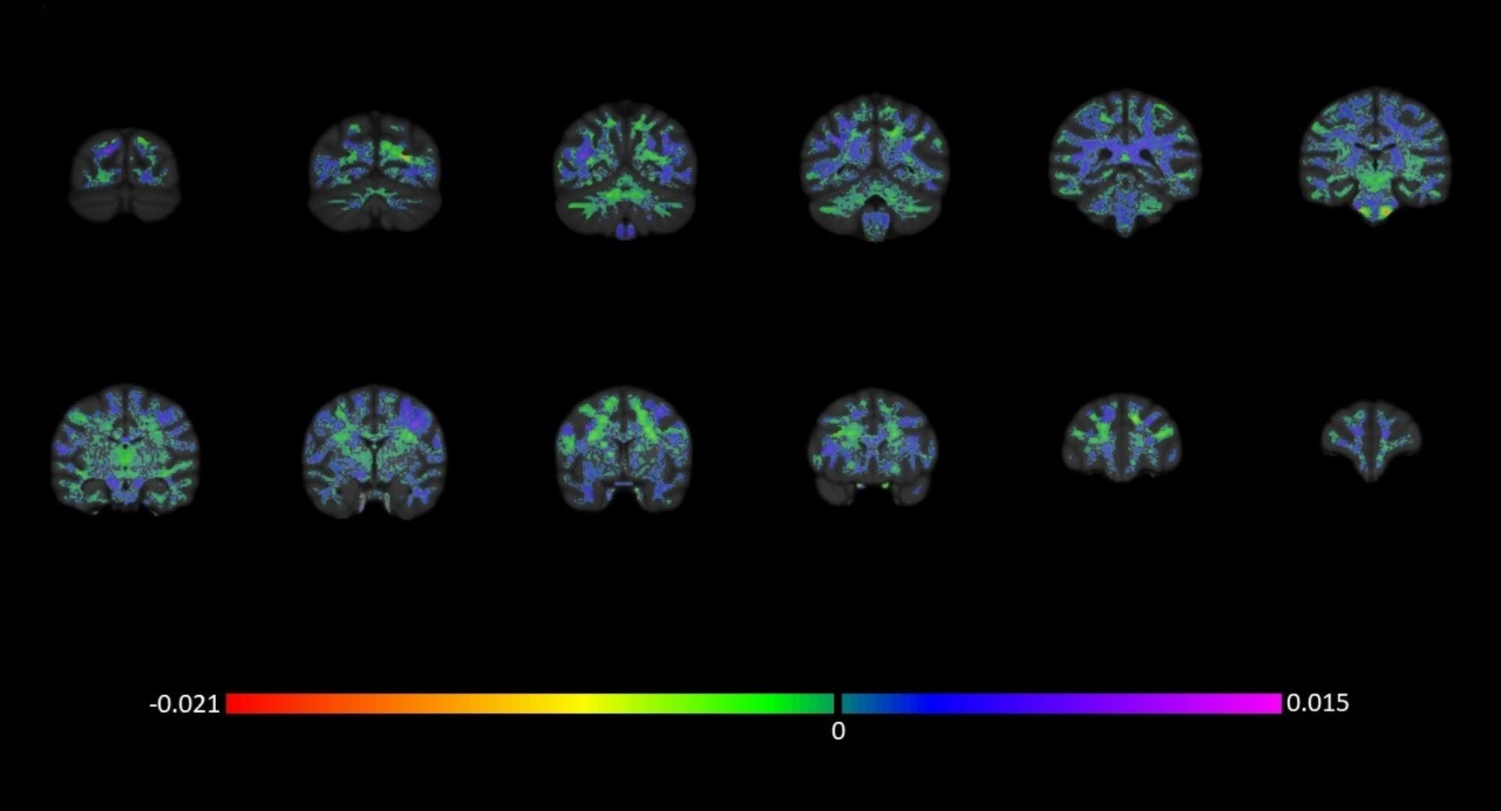
A rendering of the weight map for each white matter voxel for the whole brain machine learning analysis (coronal view; back to front). The colors indicate the relative importance of that voxel in the algorithm, with more extreme values indicating increased contribution to the decision function.

The SVM analysis revealed an overall significant proportion of chimpanzee brains were correctly classified according to rearing history (percent correct = 76.32%, *p* = 0.004, after 500 permutation tests) based on white matter FA values. The proportion of correctly classified chimpanzee brains was significant for both mother-reared (percent correct = 78.95%, *p* = 0.008) and nursery-reared subjects (percent correct = 73.68%, *p* = 0.026). As can be seen in Fig. 1, the brain regions that contributed to the decision function were widespread and found throughout the cortex. The regions contributing the most to the group discrimination include superior frontal (bilateral), rostral middle frontal (right), precentral (left), caudal middle frontal (left), brainstem (bilateral), isthmus cingulate (bilateral), posterior cingulate (bilateral), corpus collosum (bilateral), anterior/superior precuneus (left), posterior superior temporal cortex (bilateral), posterior middle temporal cortex (bilateral), lateral occipital (left), posterior precuneus (right), and cuneus (bilateral). To further explore these findings, we identified the individual chimpanzees that were misclassified and found no association between classification (correct or incorrect) and sex [*X*^*2*^(1,38) = 0.01, *p* = 0.92] or rearing history, *X*^*2*^(1,38) = 0.15, *p* = 0.70. Further, unlike our previous examination of gray matter^45^, within those who were nursery reared, there was no association between classification and previous participation in early social interventions, *X*^*2*^(1,38) = 0.19, *p* = 0.66.

**Fig. 2:**
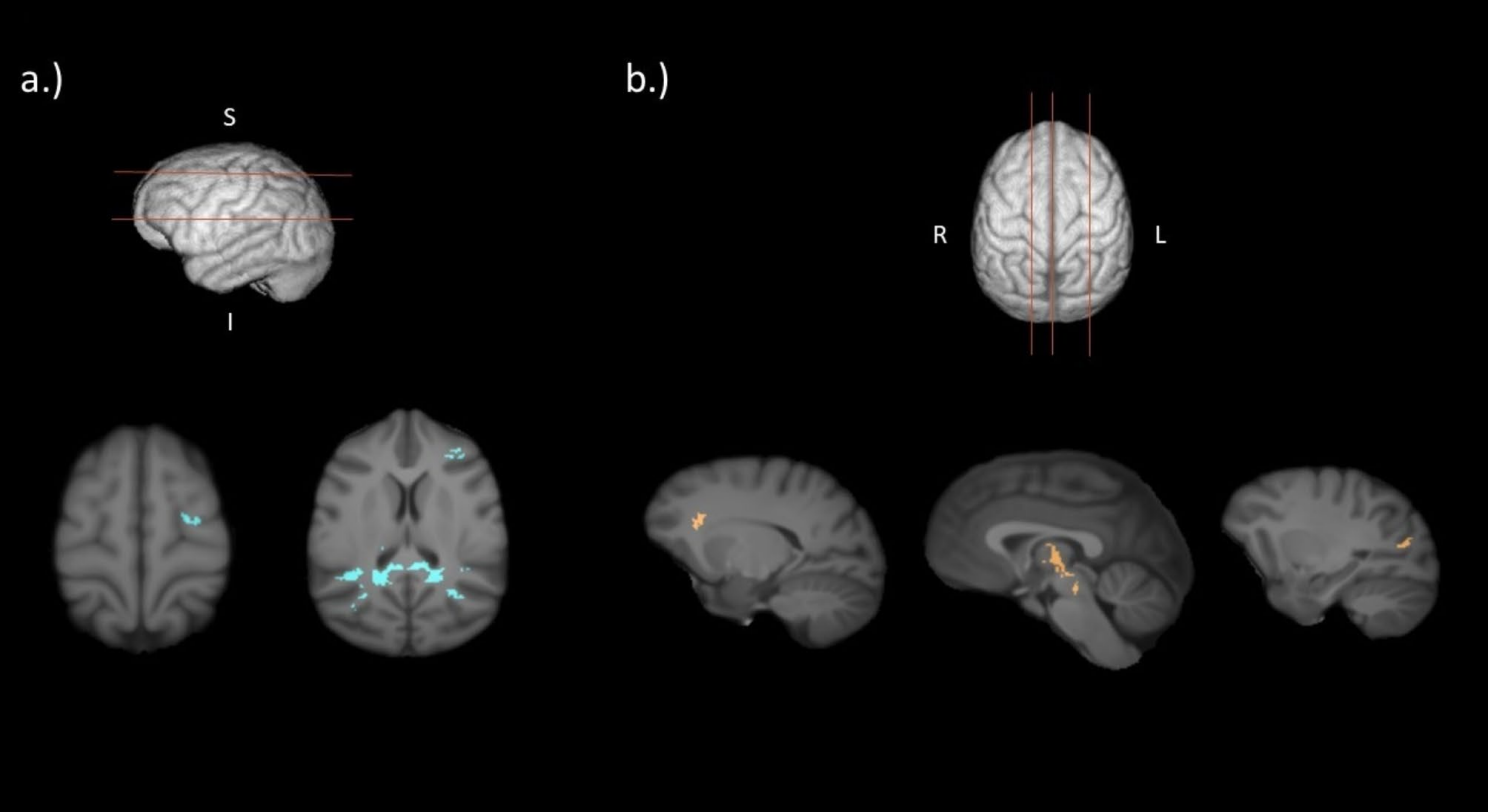
Regions of increased fractional anisotropy in (**a**) MR compared to NR chimpanzees (indicated in blue) and (**b**) NR compared to MR chimpanzees (indicated in orange) as identified by the VBM analysis (*p* < 0.01).

The VBM analysis (controlling for sex and age) revealed significant differences in white matter fractional anisotropy between mother- and nursery-reared chimpanzees (*p* < 0.01; see Fig. 2). MR chimpanzees had increased fractional anisotropy in the left and right splenium/isthmus of the corpus collosum and left premotor cortex, while NR chimpanzees had increased fractional anisotropy in the thalamic region (bilateral), left cuneus region, and right lateral genu of the corpus callosum.

## Discussion

We were able to accurately discriminate with 76.32% accuracy between mother- and nursery-reared chimpanzees based on whole-brain white matter variation using support vector machine learning. The neural differences are long-lasting, persisting into adulthood, 10–33 years after the individuals’ infant rearing experience. Further, misclassification of the nine subjects was not related to sex, rearing history, or participation in early social interventions. This was not the case in our previous study when classifying chimpanzees using gray matter variation. Nursery-reared chimpanzees who received an early social intervention (such as responsive care or language training with human caregivers, or even rearing with a heterospecific social partner) were more likely to be misclassified as mother-reared chimpanzees based on gray matter variation^45^. Together these findings suggest that rearing chimpanzees in a nursery setting has long-term effects on both gray and white matter, and that these early interventions may only ameliorate the effects on gray matter but not white matter. However, due to several limitations in the current study, more research is needed to confirm this potential differential effect on gray and white matter. First, due to the DTI data available, the current study had a smaller sample size and only included a subset of the subjects from the previous MRI study. As a result, there were fewer subjects in the current study who participated in early social interventions (*n* = 9 vs. *n* = 30; see^45^). The percentage of nursery-reared chimpanzees who participated in these interventions was 23.7% in the current study, and 35.2% in the previous MRI study^45^. Second, participation in the early interventions was not randomly assigned and intervention methods varied. One intervention, responsive care, accounts for 88.9% (*n* = 8) of intervention subjects in the current study. Responsive care was an early socioemotional intervention aimed at reducing the behavioral and attachment issues associated with standard nursery care and included additional human contact who nurtured motoric, communicative, social, and emotional species-typical behaviors over the first year (see^57^ for more details). Finally, the remaining intervention subject (*n* = 1; 11.1%) received long-term intensive communication training at the Language Research Center. This entailed 24-h care by a human caregiver for three years, intensive communication training, and socializing with other apes (see^58^ for more information on Panzee’s rearing environment). It remains for future research to randomly assign nursery-reared nonhuman primates to a single early social intervention and examine long-term effects on brain development, including differential effects on gray and white matter.

The white matter regions which produce the greatest discrimination between mother- and nursery-reared chimpanzees include the superior frontal, cingulate, corpus callosum, superior temporal cortex, and brain stem. Further, the voxel-based morphometry analysis revealed that mother-reared chimpanzees had higher white matter fractional anisotropy in the left premotor cortex as well as the splenium and isthmus of the corpus collosum, while nursery-reared chimpanzees had higher white matter fractional anisotropy in the thalamus and brainstem. Studies of chimpanzee brain development have shown that, like humans, there is prolonged development of white matter, particularly in the prefrontal cortex and corpus callosum, during the postnatal/infancy period^59–61^. Such prolonged development may make these regions more susceptible to early life experiences^61–63^, such as adverse rearing conditions. Previous studies of humans and chimpanzees found that early adverse rearing (institutional or nursery rearing, respectively) was associated with reductions in average overall white matter volume compared to controls^19,44^ even into adulthood^64^. In addition, white matter organization of institutionalized children is altered in the fronto-limbic circuitry, frontal striatal, language and sensory pathways, as well as long association fibers^5^.

Bogart et al.^44^ hypothesized that the difference in overall white matter volume and organization may be due, in part, to differences in infant nutrition. Essential fatty acids, such as docosahexaenoic (DHA) and arachidonic acids (ARA), are found in breast milk but have historically been absent or low in standard infant formulas^65^. In breast-fed infants, cortical DHA increases prenatally and shortly after birth during periods of neurogenesis, synaptogenesis, and myelination^66^. DHA also accumulates the most in oligodendrocytes which are responsible for myelination of axons throughout the brain and spinal cord^66^. Deficiencies in DHA have been associated with changes in dopamine and serotonin metabolism, altered visual attention and cognitive function, and several neuropsychiatric disorders^66,67^. However, excessive DHA supplementation can lower concentrations of ARA, another essential fatty acid, leading to reductions in birth weight, brain size, and sensory function (e.g. hearing loss), and increases in mortality and adult-onset metabolic disorders^66,68^. Pre- and postnatal supplementation with *both* DHA and ARA has been shown to improve infant developmental outcomes^66–68^. For example, in humans, supplementation with both DHA and ARA over the first year of life showed improved cognition, language, and visual acuity at 18 months^69^. In rhesus macaques, infants fed formula supplemented with both DHA and ARA, had stronger visual orienting and motor skills at an earlier age than those fed a standard infant formula^70^. Due to the importance of essential fatty acids on brain development, particularly for brain growth and myelination, these findings suggest that the standard for care of nonhuman primates reared in nursery settings should include provision of DHA and ARA supplemented formula. Although we do not have records of the formula provided to the nursery-reared chimpanzees in the current study, DHA/ARA supplemented formulas were not approved by the US Food and Drug Administration until 2002 and the nursery-reared chimpanzees were born between 1973 and 1992; therefore, it is unlikely that the formula used was supplemented with DHA/ARA. In turn, it is possible that lack of DHA/ARA contributed to the rearing group differences in white matter variation reported here. Future studies should examine any differences in white matter development in nonhuman primates that are reared in nursery settings with and without DHA supplementation.

Furthermore, this has potentially serious implications for children raised in institutional settings and, as a result, are fed infant formula. There is strong evidence of short-term effects of essential fatty acid deficiencies during childhood^67^, but the potential long-term effects (into adulthood) are unknown. Children institutionalized prior to DHA supplementation are now adults. Recent studies show that they display inappropriate social behavior, inattention, overactivity, and cognitive impairments into young adulthood^71^, as well as difficulties developing relationships, and increased health issues even later in life^72^. In addition, there is now evidence that young adults institutionalized as infants have lower total brain volume, lower overall gray and white matter volumes, as well as regional surface area and volume differences compared to non-deprived controls, despite growing up in nurturing families for the remainder of their childhood^64^. However, the cross-sectional design and absence of nutritional data in this particular study, along with the inherent confounds common to most human studies in this area, make it difficult to determine the causes and timing of brain changes, including whether or not deficiencies in DHA/ARA were involved. Well-controlled studies with animal models could help bridge this gap and isolate the relative contributions of both social rearing environments and DHA/ARA deficiencies that induce these long-term changes in the brain.

That said, once nursery-reared individuals are integrated into larger social groups there could be other social or behavioral differences that could impact these results and should be examined in the future, including quantity and quality of conspecific social interactions, social rank, and presence and/or severity of abnormal behavior. For example, social stress/subordination in rhesus monkeys during adolescence is related to overall brain volume as well as amygdala volume^73^. Additionally, increased FA in the posterior superior temporal sulcus of nursery-reared rhesus monkeys was associated with increased abnormal behavior and decreased social grooming^74^. Future studies of white matter development in differentially reared nonhuman primates should include measures of social and abnormal behavior during development.

In summary, the current study reveals long-term differences in white matter variation between nursery- and mother-reared chimpanzees, up to 33 years after their infant rearing experiences occurred. Our findings add to the existing strong evidence that early adverse rearing has long-term consequences for brain development of both human and nonhuman primates. Further, unlike gray matter, changes in white matter may not be ameliorated by early social interventions in chimpanzees. Though direct comparisons of white matter variation between those who do and do not receive early interventions are needed, the current results suggest that continued rearing of captive primates in nursery settings may lead to long-term consequences even when provided with additional early social stimulation. Future research should isolate the effects of adverse rearing and essential fatty acid deficiencies on white matter development, as well as examine social and abnormal behavior during development. Over 2.7 million children are living in institutional care globally^75^ and captive nonhuman primates continue to be reared in nursery settings; therefore, more research is needed to directly measure the long-term effects of such rearing experiences on the brain, as well as the effectiveness of combined social, cognitive, and nutritional interventions on brain development and their influence on later social behavior and cognitive functioning.

## Data Availability

All imaging data is available for download from the National Chimpanzee Brain Resource (www.chimpanzeebrain.org). Researchers may also contact Michele M. Mulholland (mmmulholland@mdanderson.org) or William D. Hopkins (wdhopkins@mdanderson.org) to request additional information or data.
